# Nutritional Aspects of Bone Health and Fracture Healing

**DOI:** 10.1155/2017/4218472

**Published:** 2017-12-31

**Authors:** Athanasios Karpouzos, Evangelos Diamantis, Paraskevi Farmaki, Spyridon Savvanis, Theodore Troupis

**Affiliations:** ^1^Department of Anatomy, School of Medicine, National and Kapodistrian University of Athens, Athens, Greece; ^2^Department of Internal Medicine, General Hospital of Athens “Elpis”, Athens, Greece

## Abstract

**Introduction:**

Fractures are quite common, especially among the elderly. However, they can increase in prevalence in younger ages too if the bone health is not good. This may happen as a result of bad nutrition.

**Methods:**

A customized, retrospective review of available literature was performed using the following keywords: bone health, nutrition, and fractures.

**Results:**

Insufficient intake of certain vitamins, particularly A and D, and other nutrients, such as calcium, may affect bone health or even the time and degree of bone healing in case of fracture. The importance of different nutrients, both dietary and found in food supplements, is discussed concerning bone health and fracture healing.

**Conclusion:**

A healthy diet with adequate amounts of both macro- and micronutrients is essential, for both decreasing fracture risk and enhancing the healing process after fracture.

## 1. Introduction

The role of nutrition in bone health is quite important. Adopting a balanced diet, rich in nutrients, minerals, and vitamins, can contribute significantly to bone health. Proper nutrition is an essential parameter of skeletal health, participating in both the prevention and the treatment of bone diseases [1]. Chronic vegetarian diet can lead to problems of osteoporosis [2]. Heredity, impaired hormonal function, pregnancy and lactation, nutrition, exercise, and various diseases (such as bronchial asthma, anorexia nervosa, and corticosteroids) affect the attainment of peak bone density [3]. If the top BMD at 25 years of age for women and 30 for men is low, then the chances of developing osteoporosis in the fourth decade of life are increased [1].

Osteoporosis can easily lead to fractures. A patient suffering from osteoporosis should have calculated the anticipated fracture risk, or this risk is enhanced. A good nutritional program can prevent osteoporosis and regulate other nutrient deficiency problems and, consequently, prevent fractures. In this article, the role of nutrition in bone health and healing is thoroughly discussed.

## 2. Methods

A customized literature search was performed focusing on the current information about the nutritional aspects of bone health and fracture healing, using the following keywords: bone health, nutrition, and fractures. Papers were included if they investigated associations between the named food groups and markers of bone health. A PubMed search for English-language, peer-reviewed, human randomized clinical trials was undertaken. Approximately 1000 articles appeared and a retrospective review was produced, after we cross-matched the sources according to their validity and impact. In conclusion up to 75 studies were included in our review.

## 3. Results

### 3.1. Calcium

An adequate calcium dietary intake, the principal component of bone, can significantly reduce the loss of bone [4]. Adequate dietary intake of calcium is necessary to replenish the compulsory daily calcium losses. In conditions of reduced intake, the body is forced to increase the process of osteolysis to maintain calcium homeostasis.

Milk and other dairy products are the best source of calcium but also significant amounts are contained in foods such as green leafy vegetables (e.g., spinach), broccoli, almonds, legumes, and seafood. The average calcium content in several foods is shown in Table 1. The recommended calcium intake varies depending on the age of the individual [4].

The absorption of calcium is about 30%. Calcium is derived from dairy and fortified foods (e.g., orange juice, tofu, and soy milk) and is nearly twice as high from certain green vegetables (bok choy, broccoli, and kale). The absorption of calcium is generally increased, when calcium is well solubilized and is inhibited in the presence of agents that bind calcium or form insoluble calcium salts. Oxalic acid and phytic acid interfere with calcium absorption and the food source containing them is considered to be a poor source of calcium. Foods with high levels of oxalic acid include spinach, collard greens, sweet potatoes, rhubarb, and beans whereas foods high in phytic acid include fiber-containing whole-grain products and wheat bran, beans, seeds, nuts, and soy isolates. The extent to which these compounds affect calcium absorption varies, and food combinations affect overall absorption efficiency [5].

### 3.2. Vitamin D

Vitamin D, also known as calciferol, comprises a group of fat-soluble secosterols and has two major forms: D_2_ and D_3_. Vitamin D_2_ (ergocalciferol) is largely human-made and added to foods whereas vitamin D_3_ (cholecalciferol) is synthesized in the skin, from 7-dehydrocholesterol, and it can be also taken dietarily via animal-based foods. They are both synthesized commercially and found in dietary supplements or fortified foods [5].

Optimal bioavailability of calcium is achieved through concomitant intake of vitamin D [3]. The administration of calcium and vitamin D supplements in later life helps reduce fractures [1]. Foods that can contain high amounts of vitamin D are eggs, liver, fish, and breakfast cereals (Table 2).

### 3.3. The Contribution of Calcium and Vitamin D in Bone Health

Administration of calcium and vitamin D supplements is now the main diet intervention against osteoporosis based on their correlation with decrease bone loss (associated with advanced age) and reduced risk of fracture [6].

Focus has been given upon the effect of increased calcium uptake, particularly in the elderly, and the addition of calcium in a low-calcium diet on reduction of skeletal bone loss [7, 8]. Nevertheless, the role of increased calcium intake, either through diet or through calcium supplements, and its correlation to hip fractures remains controversial. There has been a meta-analysis of prospective cohort studies showing no correlation between calcium intake and hip fracture risk [9]. However, there are studies such as the one of Sahni et al., which observed a protective effect of increased calcium intake on the risk of hip fracture [10].

Adequate vitamin D intake through food is a key factor in the prevention of postmenopausal bone loss. The absorption of calcium from the intestinal tract by active transport and the reabsorption of calcium by the kidney is based mainly on the action of 1,25-dihydroxycholecalciferol or calcitriol (active vitamin D). Vitamin D deficiency decreases calcium absorption from the intestinal tract and the kidneys, increases parathyroid hormone (PTH) concentration, and leads to osteolysis, which over time may lead to fracture [11]. In addition, vitamin D deficiency causes a decrease in muscle strength and increased risk of falling, increasing thus the risk of hip fracture [12].

Calcium in combination with vitamin D in dietary supplements is found to reduce the incidence of osteoporotic hip fractures. According to Van der Velde et al., the combined daily administration of calcium and vitamin D in elderly women led to a reduction in all fracture incidence by 30%, including hip fracture [13]. In an another study involving 2,686 men and women aged 65–85 years the five-year coadministration of calcium supplements and vitamin D reduced the risk of hip fracture by 33% [14]. Similar results were published in a large study of 8,124 individuals [15]. However, a study by Warensjö et al., based on the Swedish Mammography Cohort, found that low vitamin D intake reduced the rate of fracture in the first calcium quintile, but an increase in dietary calcium intake above the first quintile was not associated with further reductions in fracture risk or osteoporosis in the female population [16]. A meta-analysis by Boonen et al. suggested that oral vitamin D supplementation can reduce the risk of hip fractures only in combination with calcium supplementation [17].

Studies that focused on children's bone health did not find any association between vitamin D intake during childhood and fracture prevalence [18–20]. Only one prospective study found an association between vitamin D insufficiency in 5- to 9-year-old black children and forearm fractures [21].

### 3.4. Sodium

The relationship between sodium intake and bone health cannot be studied easily alone, as sodium interacts with other nutrients (such as potassium) and processes, such as urinary calcium excretion [22]. Excessive sodium intake, as translated by salt consumption, is a known risk factor for osteoporosis. However, a study demonstrated urinary sodium excretion and bone health are negatively correlated [23].

### 3.5. Phosphorus

Phosphorus is another component of the bone. It is located in almost all foods and by that a daily intake of 1000–1200 mg is ensured [24]. Chronic, greater than calcium, phosphorus uptake can lead to bone loss.

### 3.6. Other Nutrients That Affect Bone Health

Copper is an important component to the action of several enzymes involved in the development of interconnections between macromolecules collagen and elastin. In case of copper deficiency, disorders occur in the cartilage and bones [24]. Zinc is a cofactor for many metalloproteins involved in bone development. Incompetence during the period of growth can cause a reduction of peak bone density [18]. Manganese is a cofactor for many enzymes involved in bone metabolism [7]. Magnesium improves bone quality [25]. Reduced vitamin K levels have been associated with reduced bone density and increased risk of fractures [24]. In laboratory animals, deficiency of vitamin B6 reduced the mechanical strength of bone [4].

### 3.7. The Role of Smoking and Alcohol

Smoking and excessive alcohol consumption can adversely affect bone health. So cessation of smoking is recommended, which contributes to the overall health of the person. Regarding alcohol restriction, less than two servings of alcohol per day for men and one for women may enhance bone health [24].

## 4. Food

### 4.1. Fish

Fish with dark flesh, such as salmon, sardines, swordfish, and mackerel, are a rich source of vitamin D and are recommended as a safe way to increase the intake of vitamin D and reduce the risk of hip fracture [26]. Indeed, a study showed that fish consumption (at least once a week) was associated with a 33% reduction in the risk of hip fracture [26].

In addition, fish are also a rich source of calcium (particularly the grinding of the fish product which contains bones) and omega-3 fatty acids such as eicosapentaenoic (EPA: 20 : 5, omega-3) and docosahexaenoic (DHA: 22 : 6, omega-3). In particular fish with dark flesh contain about 2.5 grams of EPA and DHA per 100 grams [27]. Omega-3 fatty acids have the ability to lower serum concentration levels of prostaglandin E2 (PGE2) and interleukin-1 (IL-1). Both substances, mainly in laboratory studies, have demonstrated promotion of the osteolysis procedure. The constant increased intake of omega-3 fatty acids (DHA and EPA), through fish consumption, for a long time (several years) probably helps prevent hip fracture by the impact these have on bone metabolism [9].

### 4.2. Fruits and Vegetables

Some of the nutrients contained in fruits and vegetables such as potassium, magnesium, iron, and zinc have been associated with increased bone density in premenopausal women and in the elderly of both genders as well as with the reduction of bone loss in elderly men [28]. Also, a number of other nutrients such as vitamin C and niacin have been associated with increased bone density in the forearm region in postmenopausal women, while components such as proteins, phosphorus, and folic acid have been associated with reduction of postmenopausal bone loss. In a study of premenopausal women, some of the nutrients contained in fruits and vegetables such as phosphorus, potassium, magnesium, folic acid, and vitamin C were associated with increased bone mineral density of the femoral neck [29]. According to laboratory studies, the levels of serum magnesium in osteoporotic women are clearly decreased, while it has also been observed that the sponge portion of iliac bone of osteoporotic women shows less magnesium content than that of normal women [1].

Vitamin C is an essential element for the hydroxylation of bone collagen. Increased vitamin C intake, either through the consumption of fruit and vegetables or through food supplements [30], has been associated with increased bone density. The explanation of the beneficial effect of potassium, contained in fruits and vegetables, on bone density is mainly based on the theory that a diet rich in alkali acts as a safeguard to the bone by neutralizing the acid metabolites produced during the catabolism of proteins, which results in nullifying emergency release of alkali salts from the bone itself. It has been observed that potassium salts significantly improve the homeostasis of calcium and reduce bone catabolism [31]. However, substances such as potassium and vitamin C may be a simple fruit and vegetable consumption indicator. Probably, the protective effects of fruits and vegetables are due to other substances such as vitamin C which, because of its decarboxylation of osteocalcin performance, has been associated with reduced fracture risk [32].

## 5. Micronutrients

### 5.1. Vitamin A

It is well known that excessive intake of vitamin A may have very negative effects on bone health. In hypervitaminosis A, retinoic acid suppresses osteoblast activity and stimulates osteoclast formation in vitro, which leads to an increase in bone resorption and a decrease in bone formation [33]. Due to the increased absorption degree and storage of vitamin A in the body in the form of retinol, the toxicity of vitamin D may be accomplished by repeated intake of daily doses of the order of 25,000 IU to 50.000 IU [34]. Laboratory studies showed that vitamin A acts directly on osteoblasts and osteoclasts [35] causing increased bone resorption and reduced bone formation. Individual cases of hypervitaminosis A recorded occurred with bone pain and are accompanied by hypercalcemia and increased bone resorption [36]. The negative effect of vitamin A on bone health is likely due to the competitive relationship with vitamin D [34]. Thus, a study demonstrated that a dose of vitamin A contained in a liver moiety eliminates the ability of vitamin D to increase intestinal calcium absorption [37].

According to another study, women who consumed more than 1500 mg of retinol per day displayed a 10% reduction in bone mineral density of the femur and a doubling of hip fracture risk, compared with those who consumed less than 500 mg of retinol per day [38]. Also, in a prospective study of postmenopausal women, women with a daily intake of at least 2.000 mg of retinol had double risk of hip fracture compared with those with a daily intake of less than 500 mg of retinol [34]. Interestingly, a study by Lim et al. on women aged 51–74 yeas showed that up to 65% of them consume dietary supplements containing vitamin A [39]. The researchers conclude that although the risk of hip fracture was slightly increased among those consuming vitamin A supplements, no correlation was found between the above-mentioned risk and the vitamin A dose suggesting that the risk of hip fracture is not associated with the intake of vitamin A or retinol [39].

### 5.2. Vitamin K

Vitamin K has many important roles in our body. It is necessary for blood coagulation and for accumulating the data necessary for actions which potentiate the bone and help against arthritis, diabetes and wrinkles [40]. It exists in various foods but in small quantities with the exception of leafy vegetables that have dark green color. The vegetables richest in vitamin K are cabbage, spinach, beetroot, broccoli, and cauliflower.

The issue of whether there is any correlation between dietary vitamin K intake and the risk of fractures remains controversial [40]. In a cross-sectional study by Booth et al. low plasma vitamin K levels were associated with low bone mineral density at the femoral neck in men and at the spine in women without using estrogen replacements [41]. In addition, Torbergsen et al. showed that low plasma vitamin K level was associated with increased hip fracture risk [42]. More specifically for vitamin K2 supplement, a meta-analysis confirmed that its consumption can ameliorate the vertebral bone mineral density [43]. Moreover, higher levels of vitamin K are associated with elevated levels of osteocalcin; therefore, vitamin K helps the body produce osteocalcin, a protein that helps to improve bone density and reduce fracture risk [40]. Finally, data from a recent meta-analysis point out that daily intake of vitamin K is significantly associated with reduced risk of fractures [44].

### 5.3. Vitamin B_12_

Vitamin B_12_ deficiency has been associated with osteoporosis in the past. Indeed, there is, currently, a good level of evidence that low levels of vitamin B_12_ could have a significant negative impact on bones' mineral density. A recent study by Roman-Garcia et al. [45] showed that vitamin B_12_ deficiency negatively affects bone development and maintenance, a finding in line with other studies [46, 47]. However, in absence of a coherent mechanism for this effect, questions are raised regarding the role of vitamin B_12_ in bone health and the reason why B_12_ deficiency causes an increased risk of bone loss and osteoporosis. Therefore more research needs to be done in this area.

The effects of the above-mentioned micronutrients on bone health are summarized in Table 3.

## 6. Macronutrients

### 6.1. Proteins

The existence of a correlation between dietary protein intake and bone density remains doubtful (Table 4). However, dietary protein intake appears to hold an important role in growth, development, and maintenance of bone [15]. However, proteins have been implicated in causing negative homeostasis of calcium and bone loss [48]. One possible responsible mechanism is that the dietary protein increases the endogenous acid, which in turn causes the mobilization of calcium from bone stores, in order to form salts, which neutralize the existing acidity. Moreover, proteins are an essential component of bone, representing approximately 30% of bone mass, and that bone metabolism is dependent on the continuous dietary protein intake [49]. In addition, the defective protein diet is a documented causal factor of poor bone growth and maintenance, as it contributes significantly to the appearance of pathologic complications in patients with hip fracture [49].

Questions are raised regarding the relationship between the risk of hip fracture and the dietary protein intake. In a prospective study of women, the proteins of animal origin were the nutrient that was negatively associated with the risk of hip fracture [39]. The vegetable proteins do not appear to protect against the hip fracture risk. In a similar study of men and women aged 50 to 69 years, residents of Utah, USA, the dietary protein intake (both of animal and vegetable origin) was clearly correlated with risk reduction of osteoporotic hip fractures [15]. Another large study in the USA (the Nurses' Health Study) has been unable to find evidence to support the increase in the hip fracture risk of women who consume high-protein diets [50]. A prospective study of white women showed that the decrease of serum albumin, a sensitive indicator of malnutrition, was clearly associated with increased fracture risk [51]. However, there are studies to suggest a positive correlation between the intake of protein and hip fracture risk. Indeed, in a cross-sectional study the incidence of hip fracture was observed to be directly related to the per capita consumption of calcium and protein [51]. Last but not least, according to a study of osteoporotic fractures [52], elderly women following diets high in animal protein and low in vegetable protein were at increased risk of hip fracture.

### 6.2. Carbohydrates

Most studies investigating the relationship between carbohydrate and bone density have focused on fiber intake which potentially hinders the absorption of calcium and the reabsorption of estrogen from the intestinal tract. In a study, carbohydrate intake was negatively correlated with bone mineral density in the distal end of the radius [53]. However, the correlation was statistically significant only in the related monosaccharides and disaccharides. In contrast, in another study, total carbohydrate intake was associated with a reduced risk of osteoporotic fractures, including hip fracture [54] (Table 4).

### 6.3. Lipids

The dietary lipids can significantly affect bone health (Table 4). The increased intake of lipids is associated with increased fracture risk [54] and decreased bone mineral density, particularly at the lumbar spine region and the distal end of the radius. Regarding the type of lipids studies showed that both monounsaturated and polyunsaturated lipids are negatively correlated with bone mineral density of the femoral neck [11]. The consumption of saturated fats is also negatively correlated with bone mineral density in the femoral neck particularly in men older than 50 years [55]. Nevertheless, a study found that consumption of monounsaturated fats, which in the Mediterranean style diets are derived from olive oil, is associated with increased bone mineral density at the distal end of the radius in both sexes. The beneficial effects of olive oil on bone density were attributed to the high content of vitamin E [56].

There are many mechanisms that have been proposed through which the lipids could exercise their influence. One of these is by hyperinsulinemia, which may be due to increased uptake of lipids or sugars and may cause hypercalciuria, high level of magnesium in the urine, and a negative balance of calcium and magnesium. According to another mechanism, increased lipid uptake induces calcium soap which results in reduced calcium absorption. Although in healthy subjects with normal lipid-diet no interference in absorption of calcium has been observed, the type of lipid, the length of the chain, the degree of unsaturation and oxidation, and the position of the triglycerol molecule appear to affect the absorption of calcium from the intestinal tract. It has also been proposed that increased lipid uptake is accompanied by an increased intake of retinol, which causes an increase in bone resorption. However, the increased fat intake may suggest a diet low in other important nutrients, the lack of which is likely to affect the bone health [57].

## 7. Nutrition Elements Contributing to Fracture Healing

It is commonly believed that the only nutrients needed for healthy bones and, therefore, the only ones that can enhance the fracture healing process are vitamin D and calcium [58]. However, this notion does not take into account the presence of collagen, the protein that forms the bone frame, in which the calcium and other metals are deposited. The proper construction and operation of bones cannot happen without healthy collagen. Hence, a healthy bone requires not only sufficient quantities of calcium and vitamin D, but also sufficient amounts of vitamin C, lysine and proline amino acids, and other micronutrients that support the structure of collagen. The human body cannot produce vitamin C or lysine internally and consequently there is a high probability of shortage of these critical nutrients in malnutritional situations, which can be further enlarged by the stress induced by a bone fracture [58].

A randomized, double-blind, placebo-controlled clinical study, which included 131 patients aged 15–75 years with tibial fracture, evaluated the effect of supplementation with micronutrients involved in collagen building during the fracture healing time. It was observed that the group of patients treated with supplements of essential micronutrients containing vitamin C, lysine, proline, and vitamin B6 showed acceleration of fracture healing time, at 14 weeks, compared with the placebo (sugar pill) controlled arm, whose fractures healed in 17 weeks. Moreover, bone fractures in approximately 25% of patients who received the supplement healed in only 10 weeks, but only 14% of patients had the same effect in the control group. Patients receiving the supplement also reported a significant improvement in general sense of personal wellness. This study showed that healthy collagen plays an important role in optimal healing of bone fractures [58]. A simple supplementation with specific micronutrients could significantly reduce the healing time and the discomfort of patients and also reduce the financial burden on the patients and the healthcare system.

Protein seems to play a role in fracture healing. However, the protein contribution is suggested not to affect the healing process per se but to diminish the fracture consequences. A study that investigated the combined effects of protein-rich nutritional and bisphosphonate supplementation on body composition, handgrip strength (HGS), and health-related quality of life (HRQoL) following hip fracture found no differences among the groups, regarding change in fat-free mass index (FFMI), HGS, or HRQoL during the study year [59]. However, vitamin D and calcium alone preserved FFMI more effectively than the protein-rich nutrition in this relatively healthy group of hip fracture patients [59].

Malnutrition is responsible for inadequate and incomplete healing of most kinds of wounds and appears to be prevalent among the elderly. Moreover patients with hip fracture appear to be more susceptible to malnutrition than the general aging population [56]. A study evaluated the effects of different nutritional measurements on wound healing status after hip fracture in the elderly and for the purpose serum albumin, serum transferrin, serum prealbumin, and total lymphocyte count levels were used, as parameters indicative of nutritional status. According to their findings 22.2% of the patients suffered complications due to delayed wound healing that was associated with malnutrition [60]. The findings are in accordance with another study that examined the association of nutritional status as measured by the Mini-Nutritional Assessment Short Form (MNA-SF) with changes in mobility, institutionalization, and death after hip fracture. They suggested that malnutrition or risk of malnutrition as measured by the MNA-SF can be independent predictor of negative outcomes after hip fracture [61].

In a study aiming to determine the prevalence on Mini-Nutritional Assessment of protein-energy malnutrition in patients aged over 75 years admitted for hip fracture, it was found that 28% of patients suffered from malnutrition [62]. The study confirmed the high prevalence of protein-energy malnutrition in patients with hip fracture aged over 75 years, which resulted in longer hospital stay and economic burden.

Another study that evaluated the effects of nutritional supplementation with calcium, vitamin D, and bisphosphonates (alone or together) in postoperative treatment with total hip and total body bone mineral density concluded that seventy-nine patients, mostly women (71%), with a mean age of 79 years (range, 61–96 years) and with a recent hip fracture, who were living independently and were ambulatory on admission, were without severe cognitive dysfunction. The study found that protein- and energy-rich supplementation in addition to calcium, vitamin D, and bisphosphonate therapy had additive effects on total body and total hip bone mineral density among elderly patients with hip fracture [63].

Last but not least, osteoporosis may be the cause of fracture and may also inhibit fracture healing, as it is associated with osteopenia. Modifiable dietary factors, such as dietary magnesium and potassium intakes, are highly suggested to osteoporotic patients, since they are expected to have influenced bone quality due to osteoporosis, principally, via calcium-dependent alteration of bone structure and turnover [64].

## 8. Nutrition and Fracture Risk

A study in an adult population in the United Kingdom investigated the influence of dietary magnesium and potassium intakes, as well as circulating magnesium, on bone density status and fracture risk, showing that hip fracture risk was reduced both in men (*n* = 1958) and in women (*n* = 2755) with high intakes of magnesium and potassium. Furthermore, trends in fracture risk for men, among serum magnesium concentration groups, were apparent for spine fractures (*P* = 0.02) and total hip, spine, and wrist fractures (*P* = 0.02) [62].

Increased fracture risk has also been associated with vitamin K nutritional status. However, there are no consistent results supporting the role of vitamin K on bone mineral density, depending on ethnic difference and gender. A recent Korean study tried to configure this association for the Korean population, analyzing raw data from the fifth Korea National Health and Nutrition Examination Survey for adults (2,785 men, 4,307 women) aged over 19 years [65] and showing that men had a positive association between femur bone mineral density and vitamin K intake, whereas women with high vitamin K intake were associated with higher bone mineral density, both in femur and in lumber. Overall, the findings suggested an association between high bone mineral density and high dietary vitamin K intake [65]. Another study in elderly Norwegians investigated the incident risk of hip fractures, according to serum concentrations of vitamin K_1_ and 25(OH)D and it was demonstrated that low concentrations of both vitamins D and K_1_ are a significant risk factor for hip fractures [66]. However, in this study an increased risk of hip fractures was not associated with low concentrations of either vitamin alone.

A post hoc analysis assessed the association between adherence to diet quality index (developed according to dietary recommendations) or existing healthy dietary patterns and fractures in postmenopausal women [67]. The analysis used longitudinal data from 40 clinical centers throughout the United States that had been included in Women's Health Initiative (WHI) observational study. Only participants who were aged 50 to 79 years were eligible for the WHI prospective cohort which finally included 93.676 women. According to the results, a higher adherence to a Mediterranean diet is associated with a lower hip fracture risk, which concludes that a healthy dietary pattern may have a role in maintaining bone health in postmenopausal women.

Another study investigated similarities and differences between mothers and daughters, regarding dietary and nondietary risk factors for bone fractures and osteoporosis [68], examining the risk factors of 712 mothers (29–59 years) and their daughters (12–21 years) in Poland during 2007–2010. A significant correlation for calcium intake from all dairy products by mothers and daughters was found, as well as a significant correlation of the presence of bone fractures' risk factors both for mothers and for their daughters. Their results confirmed the role of family environment in bone health and demonstrated the stronger effect of negative factors of family environment, as compared to other positive factors on bone fracture risk.

The OSTPRE study [69] in Finland examined the effect of vitamin D_3_ and calcium on fracture risk in women aged 65 and 71 years, by investigating the antifracture efficacy of high dose vitamin D (800 IU) and calcium (1000 mg). 3432 women were randomized but no correlation was found between high dose intake of vitamin D_3_ and calcium and the prevention of fracture risk during the follow-up period.

The Cohort of Swedish Men (COSM) study [70] investigated the association between coffee consumption and risk of fracture among men. 42,978 men aged 45–79 years at baseline in 1997 answered a self-administered food frequency questionnaire covering coffee consumption and a medical and lifestyle questionnaire, covering potential confounders. Interestingly, no association was found between high coffee consumption and increased risk of fractures.

A prospective study in Aarhus City, Denmark, in a period of two decades investigated the association between maternal vitamin D status and offspring bone mass [71]. Although no overall association was shown between total vitamin D status and offspring bone fractures, the sensitivity analyses demonstrated a borderline significant inverse association (*P* = 0.054) between vitamin D status in pregnancy and offspring forearm fractures.

A study in Norway examined the association of vitamin K1 and 25(OH)D with an increased risk of hip fracture and whether bone turnover markers mediate the possible synergistic effect of these two micronutrients [42] and found that vitamin K1 and 25(OH)D are independently and synergistically associated with the risk of hip fracture and that this effect is possibly mediated through osteocalcin. Therefore, these two micronutrients may be important in treatment of hip fracture patients and in risk reduction of subsequent fracture, especially in patients with pronounced comorbidity.

In a British cohort study an association between a protein-calcium-potassium–rich (PrCaK-rich) dietary pattern in adult life and bone outcomes at 60 to 64 years of age was investigated [72]. The researchers used the diet diaries from the MRC National Survey of Health and Development that were collected at ages 36, 46, 53, and 60 to 64 years in 1263 participants (53,5% women). During adulthood, an increase in score for a dietary pattern rich in protein, calcium, and potassium was associated with greater size-adjusted bone mineral and volumetric bone mineral density at those sites most prone to osteoporotic fracture, the spine and the hip, thus lowering the risk of fracture at those sites.

## 9. Conclusions

Nutrition holds a dominant role in skeletal health, both in reaching the top bone density, from infancy until about the thirtieth year of life, and in maintaining bone health in the rest of adult life. A balanced diet that covers the daily caloric needs and the required daily intake of calcium and vitamin D is a key factor in achieving peak bone mass during the transition from infancy to adulthood and reducing the rate of bone loss in the elderly.

Bone density of the adult skeleton is determined by peak bone mass and bone loss rate. Studies have shown that the difference in ability to achieve peak bone mass is determined by 60–80% by genetic factors, such as ethnicity, gender, and family history, with the remaining 20–40% by environmental factors such as nutrition, exercise, habits (smoking, alcohol, and sedentary lifestyle), various diseases, and drug use [73]. In this background, a variety of food nutrients affect bone health. These components are divided into macronutrients such as proteins, carbohydrates, and fats and micronutrients such as minerals and vitamins. However, attention should be paid to the fact that there is no casual relation between different nutrients and bone health except for the case of calcium and vitamin D. For those two nutrients there is evidence of relation between fracture risk and calcium and vitamin D deficiency. A healthy diet with adequate amounts of both macro- and micronutrients is essential, for both decreasing fracture risk and enhancing the healing process after fracture.

## Figures and Tables

**Table 1:** Calcium content in different foods.

Food	Calcium concentration (mg)
Milk 3.5% or 1.5% in fat (1 cup)	290
Canned milk (1/2 cup)	329
Yoghurt (1 cup)	320
Feta cheese (30 g)	160
Gruyere (30 g)	300
Parmesan (30 g)	414
Gouda (30 g)	198
Mozzarella (30 g)	207
Cottage cheese (30 g)	135
Cheddar (30 g)	200
Edam (30 g)	207

**Table 2:** Vitamin D content in different foods.

Food	Vitamin D concentration (IUs)
Cod liver oil (1 tsp)	400
Salmon, cooked (100 g)	360
Herring, cooked (75 g)	530–699
Tuna fish, canned in oil (85 g)	200
Orange juice, fortified (1/2 cup)	50
Milk (1 cup)	103–105
Egg yolk (1 egg)	20
Beef liver, cooked (100 g)	15
Swiss cheese (30 g)	12

**Table 3:** Micronutrients and their effect on bone health.

Micronutrient	Effect on bone health
Sodium	Excessive intake is a risk factor for osteoporosis
Phosphorus	Chronic, greater than calcium intake potentially linked with bone loss
Copper	Deficiency linked with cartilage and bone disorders
Zinc	Incompetence during growth reduces peak bone density
Magnesium	Improves bone quality
Manganese	Involved in bone metabolism
Vitamin K	Low levels associated with reduced bone density and increased risk of fractures
Vitamin C	Increased intake linked with increased bone density
Vitamin A	Hypervitaminosis A causes bone resorption and decrease in bone formation
Vitamin B12	Deficiency associated with reduced bone development and maintenance

**Table 4:** Macronutrients and their effect on bone health.

Macronutrient	Effect on bone health
Proteins	Represent approx. 30% of bone mass, bone metabolism dependent on dietary protein intake
Carbohydrates	Intake is associated with reduced risk of osteoporotic fractures
Lipids	Increased intake linked with increased fracture risk
